# Poor Subjective Sleep Quality Is Associated with Poor Occupational Outcomes in Elite Soldiers

**DOI:** 10.3390/clockssleep2020015

**Published:** 2020-05-15

**Authors:** Janna Mantua, Alexxa F. Bessey, Walter J. Sowden

**Affiliations:** Walter Reed Army Institute of Research, Silver Spring, MD 20910, USA; abessey@g.clemson.edu (A.F.B.); walter.j.sowden.mil@mail.mil (W.J.S.)

**Keywords:** occupational outcomes, emotional exhaustion, functional impairment, role overload, daytime sleepiness

## Abstract

We aimed to assess the relationship between subjective sleep quality and occupationally-relevant outcomes in military personnel. Participants were from an elite unit of US Army soldiers who worked extended (~30 h) shifts (with minimal recovery time between shifts) during 3-week work sessions. Questionnaires assessing subjective sleep quality during the month prior (Pittsburgh Sleep Quality Index [PSQI]) were administered at the beginning of the session. Occupational outcomes (emotional exhaustion, functional impairment, role overload, daytime sleepiness) were assessed on the final day of the session. Regression analyses were conducted to link sleep quality and occupational outcomes. The study sample participants had relatively poor sleep prior to the exercise (PSQI Global score average = 6.3 ± 3.1). Higher PSQI Global Scores prior to the work session longitudinally predicted daytime sleepiness (*f*^2^: 0.56) after the work session. PSQI component 7, which queries daytime dysfunction attributed to poor sleep quality, longitudinally predicted emotional exhaustion, functional impairment, and role overload (*f*^2^ range: 0.19–0.70). In conclusion, poor sleep quality—in aggregation with occupationally-mandated sleep loss—is predictive of poorer subsequent occupational outcomes. Future work should aim to increase sleep opportunities prior to occupationally-mandated sleep loss in order to build resilience when sleep loss is unavoidable.

## 1. Introduction

Sleep is critical for maintaining proper psychological [1,2] and physical health [3,4]. Individuals with occupations that require extended work hours or who work during unconventional hours are negatively impacted by non-ideal sleep patterns. For instance, it is well-established that poor sleep quality and sleep loss lead to poor occupational outcomes in physicians, nurses, and first responders [5,6,7,8,9,10]. However, to date, little work has been done to investigate the impact of poor sleep quality on active duty military service members. Military occupations come with unique challenges, such as continuous operations (e.g., 36-h work shifts), physically grueling work, and situations that can lead to injury or loss of life. Furthermore, service members often work in small teams or squads in a rigid chain of command that requires formal, taxing social situations (e.g., managing lower service members, engaging with a higher-ranking member). Accordingly, individuals working for the military in operational settings are particularly susceptible to burnout, stress, and fatigue [11,12]. In the current study, we aimed to investigate the link between sleep quality and occupational functioning in an elite unit of US Army soldiers. Specifically, we examined the link between sleep quality and multiple occupational outcomes: emotional exhaustion, functional impairment, role overload, and daytime sleepiness.

### 1.1. Sleep and Emotional Exhaustion

Emotional exhaustion (a component of occupational burnout) is a chronic state of perceived psychological/emotional depletion that results from a stressful and/or overloaded occupational setting [13,14]. Higher emotional exhaustion is a strong predictor of poorer job satisfaction [15,16] and performance [17]. It is also a predictor of higher job turnover [17], more absenteeism [18] (i.e., not going to work), and higher presenteeism [19] (i.e., going to work even when one is sick, which results in lower productivity). Consequently, higher exhaustion prospectively predicts a higher rate of job turnover [17,18], a higher rate of disability claims over time [20], and even higher mortality [21]. Given the strong link between emotional exhaustion and occupational outcomes, identifying antecedents that contribute to emotional exhaustion has been an important endeavor in organizational research for nearly 40 years.

There is a strong relationship between sleep and emotion. Sleep, for instance, is critical for emotional regulation [22,23], emotional memory consolidation [24,25], and the maintenance of psychological health [1,2]. Sleep has also been linked to emotional exhaustion in the workplace. In several cross-sectional studies, emotional exhaustion was related to subjective sleep complaints and subjective sleep quality [5,26,27,28]. In one such study, sleeping less than seven hours per night was an independent predictor of emotional exhaustion, even when controlling for important factors such as exercise, anxiety, and depression [28]. Along the same lines, a separate study showed that individuals were more likely to have a decrease in emotional exhaustion longitudinally if their symptoms of exhaustion were alleviated after a period of sleep (i.e., less exhaustion in the morning than the night prior) [29]. Taken together, these findings suggest that sleep may actively play a role in exacerbating or, alternatively, recovering from feelings of emotional exhaustion. In the current study, we hypothesized that poor sleep quality would be predictive of higher emotional exhaustion.

### 1.2. Sleep and Functional Impairment

Functional impairment is a broad construct that reflects an individual’s inability to perform day-to-day duties. Definitions of functional impairment are often tailored to a specific population. For instance, older adults or individuals with physical impairment may be assessed for functionality in such activities as climbing stairs or bending over [30], while individuals with psychological disorders may be assessed for their ability to maintain employment [31]. Military service members exhibit occupational functional impairment in ways that are unique from other populations. To address this gap, the Walter Reed Army Institute of Research created and validated a scale of soldier Functional Impairment, which queries soldiers on their ability to perform their daily duties, such as completing physical tests (a daily requirement for soldiers), carrying heavy loads, and solving problems at work [32]. This questionnaire has been shown to accurately quantify active-duty soldiers’ day-to-day functioning.

In non-military settings, functional impairment has been shown to degrade with sleep loss or poor sleep quality, such that workers cannot perform their core duties efficiently after insufficient sleep. For instance, poor sleep quality has been linked with higher “decision regret” (i.e., a low satisfaction/confidence in clinical decisions) in critical care nurses [6]. Similarly, in junior resident doctors, those who worked extended shifts (i.e., those with less recovery/sleep) reported more “fatigue-related preventable adverse events” than those working standard, eight-hour shifts [33]. Further, emergency medical responders reported making more errors and engaging in a greater amount of safety-compromising behaviors following sleep loss or poor sleep [10]. Lastly, in a large-scale survey conducted by the National Sleep Foundation, individuals with sleep disorders reported a higher number of workplace impairments (e.g., problems with organization, decreased productivity, failure to finish tasks) than those with healthy sleep [34]. Despite this evidence linking sleep and functional impairment in various occupational settings, to date, there has been little research on sleep and soldiers’ functional impairment. In the current study, we tested this link and hypothesized that sleep would be predictive of greater functional impairment (i.e., inability to perform core duties).

### 1.3. Sleep and Role Overload

Role overload is a feeling of stress or overtaxing that results from a high workload with not enough time to complete the given tasks [35]. Higher role overload is predictive of poor occupational outcomes in workers from differing organizational settings. For instance, higher role overload is predictive of a greater intent to leave the workplace and a lower sense of commitment to the organization in salespersons [36], and it is also predictive of turnover intention in social workers [37]. Furthermore, women with high levels of role overload have been shown to have poorer mental health [38], and workers with role overload were more likely to have job-related physical health issues (e.g., backache, headache, stomachache, etc.) than individuals with role underload or appropriate workloads [39]. These findings indicate high role overload negatively influences worker attitudes toward their organization and negatively influences worker health and wellbeing.

There is a finite number of hours in a day, meaning people need to make decisions about what to prioritize. Consequently, there is an inverse relationship between workload and sleep duration. In fact, an indication that someone is experiencing role overload is that they reduce sleep to work longer hours [40]. Unfortunately, because less sleep leads to higher stress levels, reducing sleep duration likely increases feelings of role overload, creating a deleterious cycle. Indeed, individuals with a healthy lifestyle (i.e., who have healthy habits, such as obtaining an adequate amount of sleep) seem to be protected against role overload [41], potentially because sleep actively prevents stress levels from elevating. Although previous studies have discussed poor sleep quality and role overload as independent predictors of absenteeism [42] and burnout in nurses [43] and flight attendants [44], these factors have not been directly linked to each other in a workplace setting. In the current study, we tested whether these two factors are related, not simply concurrent. We hypothesized that soldiers with poorer sleep would have higher perceived role overload.

### 1.4. Daytime Sleepiness

Daytime sleepiness is a physiological state resulting from several wake- and sleep-promoting components: homeostatic sleep drive, circadian rhythmicity, environmental, and additional behavioral factors [45]. A reflection of daytime sleepiness is a high propensity to fall asleep when one should otherwise be awake and alert. Falling asleep in a high-risk occupational setting, of course, can be incredibly dangerous. Yet falling asleep on the job is not uncommon, especially among individuals who work in monotonous, repetitive occupations. For instance, in a sample of commercial bus drivers, 12% of the sample reported having fallen asleep at the wheel [46]. Similarly, in a sample of roughly 600 long-distance truck drivers, a startling 25% of the sample had fallen asleep at the wheel within just the year prior [47]. The consequences of falling asleep during these situations can be catastrophic, and therefore, daytime sleepiness should not be taken lightly. In military populations, who often must remain vigilant during guard duty or while supervising others, falling asleep on the job can lead to mistakes, injury, or even death.

Additionally, although falling asleep can lead to a host of unwanted consequences, excessive daytime sleepiness itself (even when an individual does not fall asleep) can similarly lead to negative outcomes. In differing occupational settings, increased daytime sleepiness has been linked to degraded work performance, such as increased work injury in factory workers [48] and increased occupational accidents in nurses [7]. It is possible that lower levels of vigilance or focus associated with high levels of daytime sleepiness can lead to poor occupational outcomes, accidents, and injuries. We believe that high levels of daytime sleepiness in a military operational setting (e.g., in training, in combat) may be dangerous, and that identifying antecedents to this factor is critical. In the current study, we tested the link between sleep quality and daytime sleepiness, and we hypothesized that poor sleep would predict higher levels of daytime sleepiness.

### 1.5. The Current Study

In sum, to date, research on the direct link between sleep and occupational functioning in military personnel has been relatively limited. Accordingly, in the current study, the first aim was to investigate the relationship between subjective sleep quality and occupational functioning in an elite unit of soldiers who are responsible for conducting high-risk training missions and who also undergo extended periods of sleep loss. We did so in an ecologically valid manner. Often, during military operations, shifting policies and procedures to accommodate more time for sleep is not feasible. Therefore, military researchers (particularly in our lab) have started focusing on increasing resiliency to sleep loss rather than eliminating sleep loss. This is done either by enhancing or extending sleep prior to sleep loss (i.e., sleep banking [49,50,51,52,53]) or by creating fatigue management strategies during and after sleep loss (e.g., using smart apps to enhance caffeine timing [54]). The current study focuses on the former concept—how does sleep quality prior to mandated sleep loss longitudinally predict the selected outcomes after sleep loss occurs? We hypothesized that better sleep quality prior to a work session containing sleep loss would predict occupationally relevant measures of performance at the end of the sleep-loss period.

## 2. Results

Overall, the sample of 35 soldiers had poor sleep on the PSQI (6.3 ± 3.1)—above the commonly-used cut-off score of 5 [54,55], consistent with prior military samples [56,57,58,59,60,61]. The participants reported obtaining 5.6 ± 1.2 h of sleep per night prior to the exercise, indicating the soldiers had a modest amount of sleep debt before beginning the exercise. Emotional exhaustion scores were high (8.9 ± 10.1), relative to population norms [14] and functional impairment scores (8.5 ± 3.5) were similar to other active-duty soldier populations [62]. Epworth Sleepiness Scale (ESS) scores were high (15.5 ± 3.9), above the excessive sleepiness cutoff score of 10.

As shown in Table 1, higher scores on the PSQI Global Score longitudinally predicted higher subsequent higher daytime sleepiness (large effect size). The PSQI Global Score did not predict emotional exhaustion, functional impairment, or role overload.

PSQI component analyses yielded different results. Specifically, for daytime sleepiness, higher (i.e., poorer) scores for component 1 (overall sleep quality), component 3 (sleep duration) and component 4 (sleep efficiency) predicted higher daytime sleepiness, both with a medium effect size. These components did not predict other occupational outcomes.

**Figure 1:**
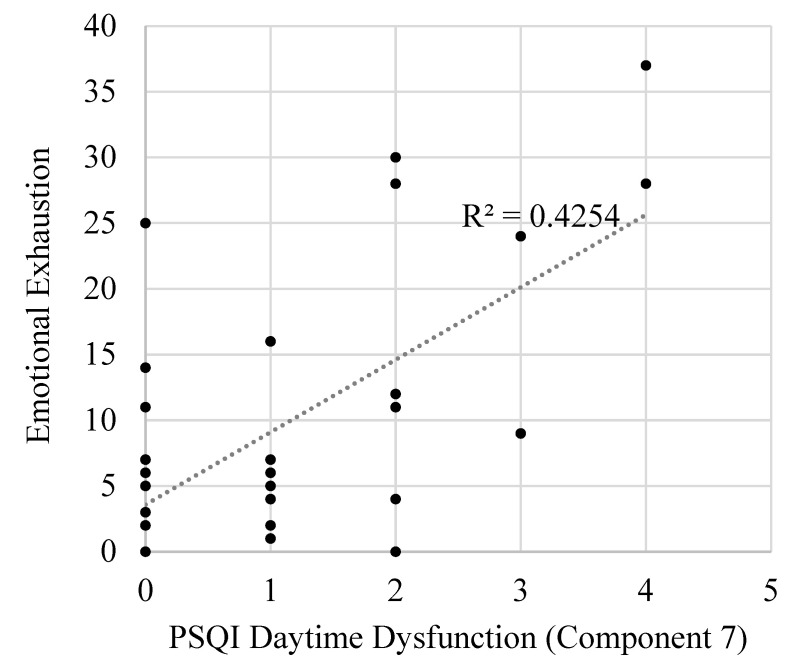
A correlation linking poor sleep quality as measured by PSQI daytime dysfunction attributed to poor sleep quality (component 7) with higher emotional exhaustion.

Conversely, component 7 (daytime dysfunction attributed to poor sleep quality) scores predicted occupational outcomes but not ESS scores. That is, component 7 predicted higher emotional exhaustion (large effect size; Figure 1), greater functional impairment (small effect size), and higher role overload (medium effect size) levels. Component 7 was not predictive of daytime sleepiness.

## 3. Discussion

Although previous studies have linked poor sleep with negative performance and psychological outcomes in active-duty military [61,63], there has been limited research on the link between sleep and relevant occupational outcomes in this unique population. The objective of this work was therefore to examine the link between sleep quality and occupational functioning in an elite unit of US Army soldiers. Specifically, we examined the link between sleep quality and multiple occupational outcomes: emotional exhaustion, functional impairment, role overload, and daytime sleepiness. This was done by measuring sleep quality before an intensive three-week work session of repeated 30-h shifts. Participants reported on their sleep quality during the month prior, providing an indication of how much theoretical sleep debt the participants had accrued prior to occupationally-mandated sleep loss. Participants then completed surveys assessing occupational outcomes after the three-week session. We found that the participants who had poorer sleep quality prior to the exercise had poorer outcomes for several— but not all—measures when the session ended. These findings are discussed in more detail below.

We hypothesized that higher PSQI Global Scores—a validated measure of sleep quality from the month prior—would longitudinally predict the tested outcome measures (emotional exhaustion, functional impairment, role overload, and daytime sleepiness) following a three-week work session that required repeated cycles of sleep loss. Our hypotheses were partially supported. First, we found that the PSQI Global Scores predicted higher daytime sleepiness. This finding is consistent with decades of previous work showing that poor quality sleep directly increases daytime sleepiness [64,65]. Yet, prior to this work, studies linking these factors had only been conducted in non-military populations, and work in military populations had been limited. For instance, a study of German soldiers showed that service members had both high PSQI scores and ESS scores before and during deployment [66]. However, a direct link between these factors was not sought. Similar findings were obtained in a Navy population [67], but, again, a direct link between these factors was not tested. Here, we found a direct, longitudinal relationship between PSQI global scores and daytime sleepiness in a military population in an operationally-relevant context. However, contrary to hypotheses, we did not find that PSQI global scores predicted other aspects of occupational functioning. Yet, interestingly, there was a link between component 7 of the PSQI, which represents deficits in daytime dysfunction attributed to poor sleep quality, and several of the outcomes. The relationship between these factors was strong and consistent, suggesting that future studies should investigate whether component 7 is a unique indicator of how sleep loss impacts soldier functioning and health.

This study is unique from work investigating sleep and occupational outcomes in other working populations. As mentioned, to our knowledge, this is the first investigation of sleep quality and a diverse set of occupational outcomes in US Army soldiers. Furthermore, this study is unique from previous studies in non-military populations because of the study design. Work from our lab (in addition to a recent systematic meta-analysis [49,50,51,52,53]) has documented the benefits of sleep banking and sleep extension prior to a period of sleep loss. That is, having good sleep quality and longer sleep duration prior to sleep loss is protective against subsequent physical and cognitive effects of sleep loss. In the current study, the PSQI queried sleep quality (i.e., cumulative sleep debt or banking) during the month prior to an intense work session that included repeated periods of sleep loss. We believe better sleep quality prior to the three-week exercise may have protected soldiers from experiencing poor occupational outcomes after—and potentially during—the exercise. These findings are in support of the hypothesis that long-term/cumulative sleep quality and quantity (rather than simply the previous night’s sleep) have lasting impacts on health and wellbeing.

### 3.1. Implications and Future Directions

There are a host of negative outcomes associated with daytime sleepiness—the factor we found to be longitudinally predicted by PSQI global scores—such as increased workplace errors [7,32]. Therefore, reducing daytime sleepiness in this population is critical. Ideally, in order to alleviate the impact of sleep on functioning, this population (and similar military populations) would be “allowed” to obtain more sleep during their operational duties. However, as mentioned, building in more sleep time is not always feasible in a military operational context due to the need for continuous operations. Furthermore, even if soldiers are able to obtain an adequate amount of sleep, environmental sleep-disruptors may degrade sleep quality [68]. We suggest that leaders encourage and build in time for sleep prior to intense work sessions so that military service members can bank sleep as a method to promote resilience. Doing so would mitigate the impact of sleep loss on health and performance and would likely promote a quicker recovery after a period of unavoidable sleep loss [49,50,51,52,53,69].

### 3.2. Study Limitations

The findings of this study should be interpreted in the context of the study limitations. Perhaps the most pressing limitation is the relatively small number of research participants included in the sample. Statistical power may have been impacted by low sample size, and null findings may be the result of a Type II statistical error (e.g., a false negative). Due to those limitations, we included effect sizes in order to aid in the interpretation of null findings [70,71]. Many of the non-significant relationships had small effect sizes, indicating the lack of statistical significance may not be due to low statistical power, but rather to no link between those factors. There are also limitations to our statistical approach, specifically when attempting to link individual PSQI components with outcome measures. The individual components of the PSQI are limited in range (most ranging from 1–3), which may have hindered our ability to detect relationships between component scores and the outcome measures. Because of this, we cannot rule out that null relationships between PSQI component scores and outcome measures of interest may simply have been due to a narrow predictive range.

Furthermore, there are methodological limitations of the study, including the use of subjective sleep measures rather than objective sleep measures (e.g., via actigraphy). Objective measures of sleep would have allowed for more discrete sleep analyses (e.g., whether sleep quantity or quality is most imperative). Future work should aim to include objective measures in order to further investigate the relationships between sleep and occupational outcomes in this population. Additionally, another limitation is that survey measures were administered only at the end of the session, not prior to the session. Ideally, surveys could have been administered at both time points, which would have allowed for the assessment of within-subject variation before and after occupationally mandated sleep loss. Future work should aim to include repeated measures in order to gain a clearer understanding of the potential causal relationship between sleep and occupationally relevant outcomes in military populations.

Lastly, there are factors that impact occupational outcomes that we could not account for in this study. For instance, we could not account for emotional labor or stress, which have previously been associated with poor occupational outcomes, particularly emotional exhaustion [72,73]. Future work should aim to include additional factors on which the link between sleep and occupational functioning can more adequately be modeled. It is also notable that there is likely a bi-directional relationship between the outcome measures and predictors reported here. For instance, several of the included factors (e.g., emotional exhaustion, role overload) disturb sleep [27,74], and thus, there may be a deleterious feedback loop between sleep loss and occupational outcomes in this population. Future work could use cross-lag longitudinal analyses to quantify the directional relationship between these factors in order to better inform interventions to improve occupational outcomes in this population.

## 4. Materials and Methods

All methods and materials were approved by the Walter Reed Army Institute of Research Institutional Review Board (IRB) in July 2018. Participants provided written informed consent. 

### 4.1. Participants

Participants were from an elite unit of soldiers responsible for training other soldiers on leadership skills and simulating mock-combat situations in mountainous terrain. During these activities, soldiers must train and evaluate students while also ensuring that students are kept safe in the precarious environment. At the same time, soldiers must undergo physically grueling work, such as traversing mountains. Soldiers must complete these tasks while undergoing cycles of sleep loss (described further below).

Thirty-five male soldiers (age 32.1 ± 4.7 years) participated in the study. The soldiers were, on average, relatively advanced in their careers. There were no junior enlisted soldiers (E1−E4), 19 junior non-commissioned officers (E5−E6; 42%), 20 senior non-commissioned officers (E7-E9; 45%), and six officers (O1−O3; 13%). The majority of the sample had a high school education/General Education Development (GED) (*n* = 17; 38%) or a college/associate’s degree (*n* = 19; 42%). There were fewer participants with a bachelor’s degree (*n* = 7; 16%) and two with a graduate degree (4%). The average body mass index of soldiers was 26.4 ± 3.0 kg/m^2^.

### 4.2. Study Design

These data are a part of a longitudinal study designed to test the impact of sleep loss on soldier health and wellness. The current data are from two time points—one that occurred before a three-week class session and one that occurred after. During the class session, soldiers worked extended overnight shifts (from roughly 0400 to 1000 on the following day, totaling around 30 h) to train students. During each 30-h shift, soldiers may have obtained up to 1–3 h of sleep, if the opportunity presented itself. Following the 30-h shift, soldiers had about 16 h to recover in their homes before working another shift.

Soldiers were given a brief questionnaire packet containing the surveys (described below) on the first and last day of the three-week session. On the first day, participants completed the PSQI (referencing sleep during the month prior to the exercise). On the last day, they completed questionnaires querying emotional exhaustion, functional impairment, role overload, and daytime sleepiness. Additional measures on team-based military-relevant factors (e.g., team cohesion, leadership effectiveness) were included as well, but were not deemed relevant in these analyses.

### 4.3. Survey Measures

Subjective sleep quality: The Pittsburgh Sleep Quality Index (PSQI) [54,55] is a multi-component measure of sleep quality that queries participants on sleep duration, sleep onset latency, causes of awakenings, and functional outcomes related to sleep loss. The seven components created from these data are summed to create a PSQI Global Score (described in statistical analyses section). A higher number indicates poorer sleep quality.

Emotional exhaustion: The Emotional Exhaustion Scale, which is a subscale from Maslach’s Burnout Inventory [13] that can be used in isolation [17,18], queries the level of emotional exhaustion or depletion felt by participants. A higher number indicates greater dysfunction.

Soldier-specific functional impairment: The Walter Reed Functional Impairment Short Scale is a military-relevant scale assessing how well participants feel they can carry out their occupational duties [32]. A higher number indicates greater dysfunction.

Role overload: Role overload was assessed using a three-item scale from the Michigan Organizational Assessment Questionnaire [35], which asks whether participants feel they are able to accomplish all that is required of them through their occupational role. A higher number indicates greater dysfunction.

Daytime sleepiness: Daytime sleepiness was assessed using the Epworth Sleepiness Scale (ESS) [75]. The ESS asks participants how likely they are to fall asleep in a number of common scenarios. A higher number indicates greater dysfunction.

### 4.4. Statistical Analyses

Descriptive statistics were conducted by calculating mean ± standard deviation.

For the main set of analyses, we conducted a series of linear regressions between subjective sleep quality from the month prior (PSQI Global Scores) and occupational outcomes (emotional exhaustion, functional impairment, role overload, daytime sleepiness), controlling for age and military rank. Military rank was used as a covariate because certain responsibilities (e.g., managing other soldiers) tend to increase with higher rank, thereby increasing occupational stress.

Next, component scores from the PSQI were used as predictors (component 1, overall sleep quality = item #9 score; component 2, sleep latency = #2 and #5a; component 3, sleep duration = #4 score; component 4, sleep efficiency = total hours of sleep/total hours in bed; component 5, sleep disturbance = #5b–5j; component 6, sleep medication = # 6 score; component 7, daytime dysfunction attributed to poor sleep quality = #7 + #8).

Because of the relatively small sample size, effect sizes (*f*^2^) were calculated for each analysis, in accordance with Cohen’s models [70,71]) in order to aid in the interpretation of null findings.

## Figures and Tables

**Table 1:** Regression analyses assessing the link between sleep quality (Pittsburgh Sleep Quality Index (PSQI) scores) and occupational outcomes. EE = emotional exhaustion; FI = functional impairment, RO = role overload, DS = daytime sleepiness. CI = confidence interval. Bold indicates a statistically significant relationship between the two listed factors (*p* < 0.05). Cohen’s *f*^2^ of 0.10 = a small effect, 0.25 = a medium effect, and 0.40 = a large effect.

Component	Predictor	Outcome	B	Lower CI	Upper CI	R^2^	*p*	*f* ^2^
--	Global Score	EE	0.49	−0.99	1.98	0.09	0.06	0.10
		FI	0.26	−0.34	0.87	0.05	0.38	0.05
		RO	0.25	−0.19	0.68	0.18	0.13	0.22
		DS	0.86	0.33	1.39	0.36	0.017	0.56
1	Overall Sleep Quality	EE	2.75	−3.51	8.99	0.10	0.21	0.11
		FI	0.55	−1.98	3.01	0.02	0.79	0.02
		RO	−0.61	−2.75	1.53	0.04	0.57	0.04
		DS	3.36	0.84	5.87	0.26	0.026	0.35
2	Sleep Latency	EE	−2.33	−5.42	0.74	0.14	0.22	0.16
		FI	0.06	−1.23	1.35	0.01	0.43	0.01
		RO	−0.10	−1.21	1.00	0.03	0.81	0.03
		DS	0.63	−0.90	2.17	0.06	0.64	0.06
3	Sleep Duration	EE	1.20	−2.38	4.80	0.08	0.18	0.09
		FI	0.60	−0.84	2.03	0.04	0.92	0.04
		RO	0.65	−0.57	1.87	0.06	0.27	0.06
		DS	1.84	0.38	3.30	0.23	0.031	0.30
4	Sleep Efficiency	EE	−3.12	−7.66	1.42	0.14	0.49	0.16
		FI	0.83	−0.90	2.55	0.08	0.50	0.09
		RO	0.33	−1.10	1.75	0.12	0.47	0.18
		DS	2.17	0.36	3.98	0.23	0.004	0.30
5	Sleep Disturbance	EE	4.48	−2.20	11.17	0.12	0.25	0.18
		FI	−0.26	−3.03	2.51	0.01	0.85	0.02
		RO	−0.01	−2.40	2.39	0.03	0.72	0.03
		DS	−0.03	−3.19	2.12	0.03	0.47	0.03
6	Sleep Medication	EE	3.58	−2.39	9.53	0.11	0.27	0.12
		FI	2.08	−0.24	4.40	0.12	0.20	0.14
		RO	0.26	−1.89	2.42	0.03	0.80	0.03
		DS	−0.96	−3.80	1.86	0.05	0.43	0.05
7	Daytime Dysfunction	EE	3.43	1.71	5.16	0.41	<0.001	0.70
		FI	1.54	0.13	2.95	0.16	0.003	0.19
		RO	1.50	0.32	2.67	0.22	0.019	0.28
		DS	0.22	−0.77	1.22	0.04	0.79	0.04

## Abbreviations

PSQI	Pittsburgh Sleep Quality Index
EE	Emotional exhaustion
FI	Functional impairment
RO	Role overload
DS	Daytime sleepiness
